# Pharmacological Characterization of P626, a Novel Dual Adenosine A_2A_/A_2B_ Receptor Antagonist, on Synaptic Plasticity and during an Ischemic-like Insult in CA1 Rat Hippocampus

**DOI:** 10.3390/biom13060894

**Published:** 2023-05-27

**Authors:** Martina Venturini, Federica Cherchi, Clara Santalmasi, Lucia Frulloni, Ilaria Dettori, Daniela Catarzi, Felicita Pedata, Vittoria Colotta, Flavia Varano, Elisabetta Coppi, Anna Maria Pugliese

**Affiliations:** 1 Department of Neuroscience, Psychology, Drug Research and Child Health (NEUROFARBA), Section of Pharmacology and Toxicology, University of Florence, 50139 Florence, Italy; martina.venturini@unifi.it (M.V.); federica.cherchi@unifi.it (F.C.); clara.santalmasi@unifi.it (C.S.); lucia.frulloni@unifi.it (L.F.); ilaria.dettori@unifi.it (I.D.); felicita.pedata@unifi.it (F.P.); elisabetta.coppi@unifi.it (E.C.); 2 Department of Neuroscience, Psychology, Drug Research and Child Health (NEUROFARBA), Section of Pharmaceutical and Nutraceutical Sciences, University of Florence, 50019 Sesto Fiorentino, Italy; daniela.catarzi@unifi.it (D.C.); vittoria.colotta@unifi.it (V.C.); flavia.varano@unifi.it (F.V.)

**Keywords:** CA1 neurotransmission, hippocampal slices, cerebral ischemia, paired-pulse facilitation, anoxic depolarization, adenosine signaling, adenosine ligands

## Abstract

In recent years, the use of multi-target compounds has become an increasingly pursued strategy to treat complex pathologies, including cerebral ischemia. Adenosine and its receptors (A_1_AR, A_2A_AR, A_2B_AR, A_3_AR) are known to play a crucial role in synaptic transmission either in normoxic or ischemic-like conditions. Previous data demonstrate that the selective antagonism of A_2A_AR or A_2B_AR delays anoxic depolarization (AD) appearance, an unequivocal sign of neuronal injury induced by a severe oxygen-glucose deprivation (OGD) insult in the hippocampus. Furthermore, the stimulation of A_2A_ARs or A_2B_ARs by respective selective agonists, CGS21680 and BAY60-6583, increases pre-synaptic neurotransmitter release, as shown by the decrease in paired-pulse facilitation (PPF) at Schaffer collateral-CA1 synapses. In the present research, we investigated the effect/s of the newly synthesized dual A_2A_AR/A_2B_AR antagonist, P626, in preventing A_2A_AR- and/or A_2B_AR-mediated effects by extracellular recordings of synaptic potentials in the CA1 rat hippocampal slices. We demonstrated that P626 prevented PPF reduction induced by CGS21680 or BAY60-6583 and delayed, in a concentration-dependent manner, AD appearance during a severe OGD. In conclusion, P626 may represent a putative neuroprotective compound for stroke treatment with the possible translational advantage of reducing side effects and bypassing differences in pharmacokinetics due to combined treatment.

## 1. Introduction

In recent years, the use of multi-target compounds has gained the interest of the scientific community considering their several advantages (i.e., eliminating the risk of drug–drug interactions, reduction of possible side effects, pharmacokinetics and metabolism) in the treatment of various pathological conditions, such as cerebral ischemia [1,2]. Ischemic stroke is a leading cause of permanent disability and death worldwide today [3]. Although much pharmacological progress has been made in the field, current treatments are still limited by a very narrow therapeutic time window, i.e., within 4 h from the insult for the thrombolytic enzyme tissue plasminogen activator (tPA), and several side effects. Therefore, the study of new possible treatments for cerebral ischemia is extremely necessary, especially given the widening of the therapeutic time windows with new pharmacological tools preferably compatible with the current stroke treatments [4,5].

It is known that adenosine, an endogenous neuromodulator, whose concentration under control normoxic conditions is about 200 nM [6,7], is massively released during an ischemic episode reaching micromolar concentrations [6,8]. Adenosine effects are mediated by activating four receptor subtypes, namely A_1_, A_2A_, A_2B,_ and A_3_ adenosine receptors (A_1_AR, A_2A_AR, A_2B_AR, and A_3_AR) [9]. Adenosine shows different affinity versus its receptors: high-affinity A_1_AR, A_2A_AR, and A_3_AR subtypes are activated by nanomolar concentrations of adenosine (EC_50_ = 1–10 nM, 20 nM, and 300 nM, respectively [10]) whereas, at variance, low-affinity A_2B_ARs need micromolar concentrations (EC_50_ = 5–50 µM, [10]) to be recruited. The involvement of adenosine receptors in synaptic plasticity phenomena is well described in the hippocampal region, which is also a brain area particularly susceptible to a hypoxic-ischemic event [11,12,13]. Paired-pulse facilitation (PPF) is a well-established model of short-term synaptic plasticity and is considered an index of the probability of neurotransmitter release. It is known that the activation of Gi-coupled A_1_ARs, in the CA1 region of the hippocampus, increases the PPF ratio due to a reduction of neurotransmitter release [14]. On the contrary, the stimulation of A_2A_ARs or A_2B_ARs, coupled with Gs proteins, reduces PPF thus facilitating neurotransmitter release [15,16]. Since in the CA1 hippocampal region it is known that fEPSP slope depends mostly on glutamatergic transmission [17] and that PPF decrease reflects an increase in neurotransmitter release [18], it appears that selective stimulation of either A_2A_AR or A_2B_AR enhances glutamate release in this brain region, as already mentioned [15,16,19]. Of note, previous data demonstrated that either A_2A_AR- and A_2B_AR-mediated effects in the CA1 hippocampus depend on the activation of A_1_ARs since they are prevented in the presence of the selective antagonist, DPCPX [15,20].

The increase in glutamate release plays different roles under physiological conditions, facilitating neuronal excitability, synaptic plasticity (i.e., LTP), and coordination of neural networks. However, under pathological conditions (such as cerebral ischemia), an increase in glutamate release contributes to excitotoxic damage. This results from glutamatergic N-methyl-D-aspartate (NMDA) receptor over-activity, which leads to an excessive rise in cytoplasmic Ca^2+^ to neurotoxic levels and triggers the activation of many enzymes that cause acute excitotoxic cell death [21,22]. In an in vitro model of cerebral ischemia, obtained by oxygen and glucose deprivation (OGD), the over-activity of NMDA receptors is strictly correlated to the appearance of anoxic depolarization (AD), a clear sign of neuronal injury [23,24]. The AD propagates from the ischemic core to the surrounding area, called penumbra, which for decades is defined as the crucial battlefield of cerebral ischemia [25,26,27,28]. Indeed, the penumbra is a brain tissue that undergoes hypoperfusion but preserves transmembrane electrical activity [28,29]. Therefore, it represents a brain region potentially salvageable, and it is recognized that the application of a pharmacological treatment that delays AD results neuroprotective in preserving brain tissue following an ischemic-like insult [23,30,31,32].

On these bases, many studies have been conducted on A_1_AR activation that revealed a protective role of these receptors during cerebral ischemia. Unfortunately, the use of A_1_AR agonists has numerous side effects and their development has been stalled. Concerning the A_3_AR, there are currently some contradicting results in the literature about the function of these receptor subtypes in the pathophysiology of cerebral ischemia [9,31,33,34]. Therefore, the research focus has shifted to the “A_2_AR” subtypes, A_2A_ARs and A_2B_ARs that share the same Gs-coupled intracellular pathway, even if they can also activate several different transducing pathways to afford neuroprotection in dangerous brain conditions [35]. Unlike the A_2A_ARs, the A_2B_ARs have low affinity for the endogenous ligand and are scarcely, but uniformly expressed [36] in the hippocampus [37]. It is known that the selective A_2A_AR or A_2B_AR antagonism delays or prevents AD appearance during a severe OGD in the CA1 region of rat hippocampus [30,38]. However, the development of new multi-target ligands needs to be deepened to find novel therapeutic strategies for complex diseases, such as cerebral ischemia, to increase the therapeutic time window and minimize possible side effects. In this study, we provide the first functional characterization of a newly synthesized dual A_2A_AR/A_2B_AR antagonist, P626, on PPF and OGD conditions in the CA1 area of the rat hippocampus as an advantageous therapeutic approach to dampen neurodegeneration after energy failure in the brain.

## 2. Materials and Methods

All animal experiments were carried out according to the Italian Law on Animal Welfare (DL 26/2014). The document was approved by the Italian Ministry of Health (authorization code: 301/2021) and by the Institutional Animal Care and Use Committee of the University of Florence. To minimize animal suffering for our experiments we used only the number of animals necessary to obtain consistent scientific results. Male Wistar rats (Envigo, Italy, 100–150 g body weight for PPF experiments; 150–180 g body weight for OGD experiments, 6–8 weeks old) were used. All animals were located in a temperature-controlled room (22  ±  1 °C) in groups of two-five per cage, with food and water ad libitum, and with a 12 h light/dark cycle.

### 2.1. Preparation of Acute Hippocampal Slices

All the experiments were performed on hippocampal slices, acutely isolated from rat brains, as already described [31,39]. Rats were anesthetized with isoflurane (Baxter, Rome, Italy) and then sacrificed by decapitation. The hippocampi were quickly removed and placed in ice-cold oxygenated (95% O_2_–5% CO_2_) artificial cerebrospinal fluid (aCSF) of the following composition (mM): NaCl 125, KCl 3, NaH_2_PO_4_ 1.25, MgSO_4_ 1, CaCl_2_ 2, NaHCO_3_ 25, and D-glucose 10. Transverse slices (400 μm nominal thickness) were cut using a McIlwain Tissue Chopper (Mickle Laboratory Engineering Co. Ltd., Gomshall, UK) and kept in oxygenated aCSF at room temperature to recover their functionality for at least 1 h. Once this time lapsed, a slice was transferred on a nylon mesh, completely submerged in a small chamber (0.8 mL), and superfused with oxygenated aCSF (31–32 °C) at a constant flow rate of 2 mL/min. The treated solutions reached the preparation in 60 s and this delay was considered in our calculations.

### 2.2. Extracellular Recordings

**Figure 1:**
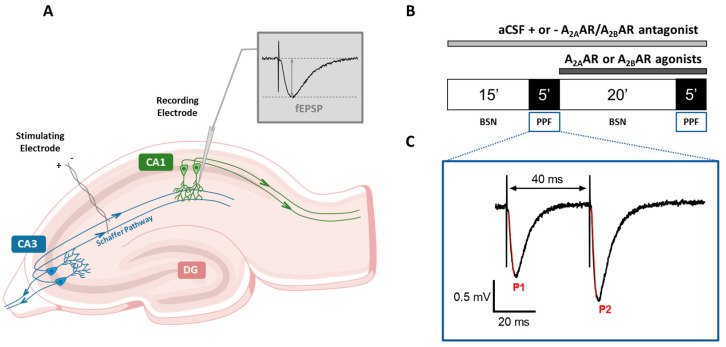
Experimental procedure. (**A**) Schematic representation of a hippocampal slice showing the synaptic circuits (DG: dentate gyrus; CA3: Cornu Ammonis 3; CA1: Cornu Ammonis 1) and the localization of the stimulating and recording electrodes. (**B**) Schematic diagram indicating the protocol utilized for drugs (A_2B_AR or A_2A_AR ligands) application. BSN: basal synaptic neurotransmission, PPF: paired–pulse facilitation. (**C**) Representative of a double field excitatory post–synaptic potential (fEPSP) response elicited by a PPF protocol (40–ms interval) in a typical experimental procedure. P1: first fEPSP; P2: second fEPSP.

A bipolar nichrome electrode was located in the CA1 stratum radiatum to stimulate the Schaffer collateral-commissural fibers with test pulses (80 μs, 0.066 Hz) delivered every 15 s. Evoked potentials were extracellularly recorded with borosilicate microelectrodes (2–10 MΏ, Harvard Apparatus Ltd., Edenbridge, UK) filled with 150 mM NaCl. The recording electrode was situated at the CA1 dendritic level to record field excitatory postsynaptic potentials (fEPSPs, Figure 1A). Data were amplified (200×, BM 622, Mangoni, Pisa, Italy), digitized (sample rate, 33.33 kHz), and stored for later analysis with LTP (version 2.30D) program [40]. Synaptic potentials were expressed as the initial slope (calculated between 20 and 80% of maximal amplitude). Input-output curves were constructed by gradual increases in stimulus strength at the beginning of each experiment. To generate a synaptic response of about 40% of the maximum, we adjusted the test stimulus strength, and it was kept constant throughout the experiment. The onset of each experiment was established after recording a stable baseline for 30 min.

### 2.3. Paired-Pulse Facilitation

Paired-pulse facilitation (PPF) was obtained by stimulation of Schaffer collateral-commissural fibers twice (40 ms inter-stimuli interval). We chose the 40 ms interstimulus interval because, as also reported in the literature [18], this value is particularly useful to underline the eventual effect, of a given compound, on presynaptic neurotransmitter release as it induces a robust potentiation of the second fEPSP over the first. After steady control baseline responses were established (basal synaptic neurotransmission: BSN), the PPF protocol was applied, still once every 15 s, for 5 min either before or after 20 min application of the selected compounds (see Figure 1B,C). The degree of facilitation was calculated as the PPF ratio (PPR) between the slope of the second (P2) and the first (P1) fEPSPs (P2/P1; Figure 1B,C). 

### 2.4. Oxygen-Glucose Deprivation

OGD insults in vitro were realized by superfusing the slice with aCSF without glucose and oxygen, and gassed with nitrogen (95% N_2_–5% CO_2_) [30,41] for 30 min. This OGD-time duration does not allow the recovery of fEPSPs as it always induces irreversible synaptic failure, as previously demonstrated by us [30,42]. After the OGD insult, each slice was again superfused with normal, glucose-containing, oxygenated aCSF. The new mixed A_2A_AR/A_2B_AR antagonist, P626, was applied 15 min before, during, and 5 min after OGD. In all conditions, fEPSPs were continuously monitored and never recovered their amplitude after a 30 min OGD, in line with our previous results [30,42]. In some experiments, both the amplitude and initial fEPSP slope were quantified, but since no appreciable differences between these two parameters were observed in drug effects nor during OGD, we calculated only the amplitude measurement (data not shown). AD was recorded as negative extracellular direct current (d.c.) shifts induced by OGD. This phenomenon is considered a sign that the cells around the tip of the glass electrode were depolarized [43]. AD latency was calculated from the beginning of OGD insult and was expressed in min; while AD amplitude was calculated at the maximal negativity peak and expressed in mV. In this work, AD amplitude values were expressed as positive values.

### 2.5. Drugs

**Figure 2:**
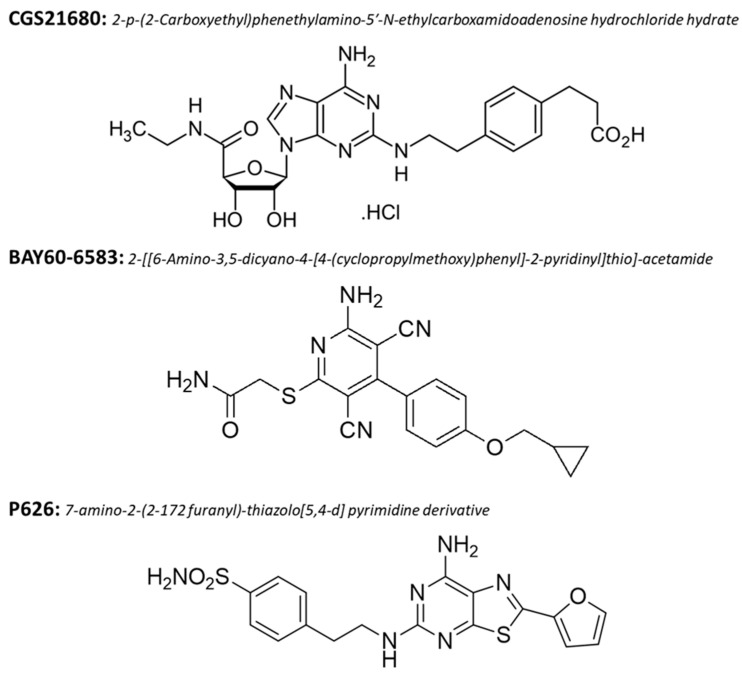
Chemical structures of the selective A_2A_AR and A_2B_AR agonists (CGS21680 and BAY60-6583, respectively) and of the new dual A_2A_AR/A_2B_AR antagonist, P626.

We used the prototypical A_2B_AR agonist 2-[[6-amino-3,5-dicyano-4-[4-(cyclopropylmethoxy) phenyl]-2-pyridinyl] thio]-acetamide (BAY60-6583, Figure 2), and the prototypical A_2A_AR agonist [2-p-(2-carboxyethyl) phenenethylamino-5′-N-ethylcarbossiamideadenosine hydrochloride hydrate] (CGS21680, Figure 2). Both compounds were purchased from Tocris (Bristol, United Kingdom). The new dual A_2A_AR/A_2B_AR antagonist, the 7-amino-2-(2-furanyl)-thiazolo [5,4-d] pyrimidine derivative (P626, Figure 2), was synthesized by Varano et al. (compound 2 in Varano et al., 2020 [44]). P626 showed high potency at both hA_2A_AR and hA_2B_AR (IC_50_ = 5.20 nM and 34 nM, respectively, cAMP assay). P626 showed a Ki = 1326 nM at hA_1_AR and a Ki = 1874 nM at hA_3_AR. All drugs were dissolved in dimethyl sulphoxide (DMSO). Stock solutions of 1000–10,000 times the desired final concentration were stored at −20 °C. The final concentration of DMSO (0.05% and 0.1% in aCSF) used in our experiments did not affect either fEPSP slope or amplitude in all different protocols applied.

### 2.6. Statistical Analysis

Data were expressed as mean ± SEM (standard error of the mean). Kolmogorov–Smirnov normality test was performed to check data distribution: all data reported in the present research are normally distributed. Two-tailed Student’s paired or unpaired *t*-tests or one-way ANOVA followed by Bonferroni post-test analysis were performed, as appropriated, in order to determine statistical significance (set at *p* < 0.05) between groups. Data were analyzed using “GraphPad Prism” (GraphPad Software, San Diego, CA, USA) software.

## 3. Results

In this study, we functionally characterized the new mixed A_2A_AR/A_2B_AR antagonist, P626, in the CA1 region of rat hippocampus, a brain area involved in synaptic plasticity phenomena and particularly susceptible to hypoxic-ischemic injuries. All data were obtained by an extracellular recording of fEPSP from 85 slices isolated from 37 rats.

### 3.1. The New A_2A_AR/A_2B_AR Antagonist, P626, Prevented the Effects of Selective A_2A_AR or A_2B_AR Agonists on PPF in the CA1 Hippocampal Slices

**Figure 3:**
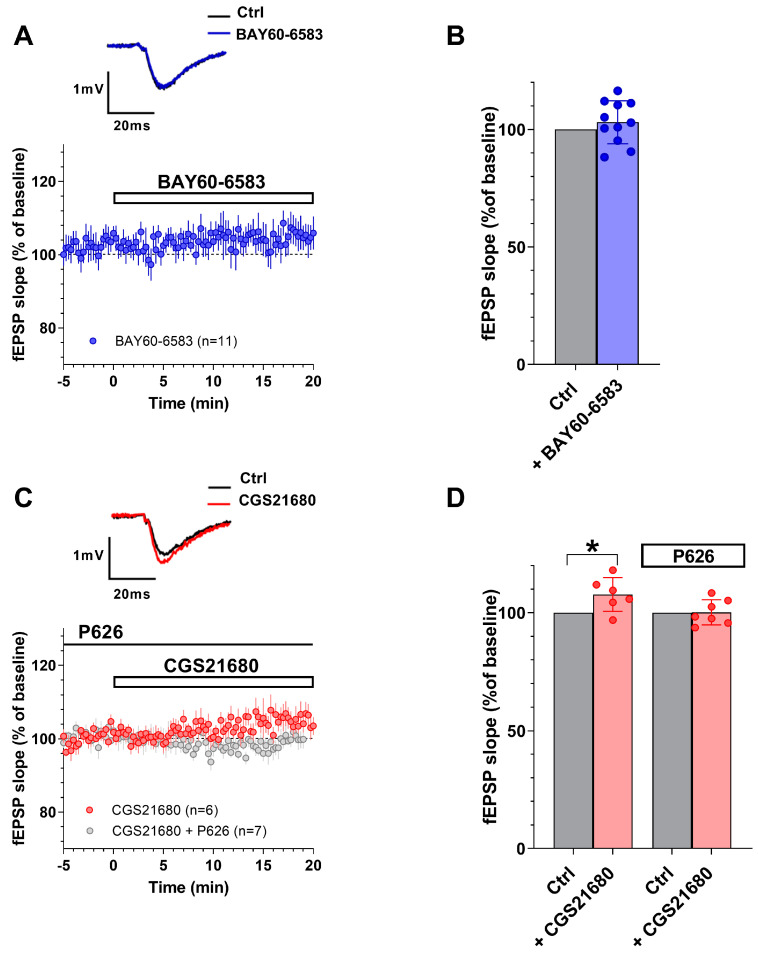
The new dual “A_2_ARs” antagonist, P626, prevented the increase of basal synaptic transmission induced by the selective A_2A_AR agonist CGS21680 in the CA1 region of the rat hippocampus. (**A**) Averaged time course of fEPSP slope under basal synaptic transmission, before and during applying the selective A_2B_AR agonist BAY60–6583 (200 nM, *n* = 11 slices taken from 10 animals). Insert: original fEPSPs traces recorded in a typical experiment before (Ctrl, black trace) and after 20 min BAY60–6583 (blue trace). (**B**) Pooled data of fEPSP slope (mean ± SEM), expressed as a percentage of respective baseline measured 5 min before and during the last 5 min of the application of BAY60–6583. (**C**) Averaged time course of fEPSP slope under the basal synaptic transmission, before and during the application of the selective A_2A_AR agonist CGS21680 (50 nM, *n* = 6 slices taken from 5 animals, red circles) applied alone or in the presence of P626 (200 nM, *n* = 7 slices taken from 5 animals, grey circles). Insert: original fEPSPs traces recorded in a typical experiment before (Ctrl, black trace) and after 20 min CGS21680 application (red trace. (**D**) Pooled data of fEPSP slope (mean ± SEM), expressed as a percentage of respective baseline measured 5 min before and during the last 5 min of the application of CGS21680 alone or in the presence of P626. Note that CGS21680 significantly enhanced basal synaptic transmission and that this effect was antagonized in the presence of P626. Paired columns refer to data collected from the same slice, before (Ctrl) or after selective agonist application (BAY60–6583 in (**B**) and CGS21680 in (**D**)). * *p* < 0.05 vs. respective Ctrl, paired Student’s *t*–test.

In a first series of experiments, we tested the effects of the selective A_2B_AR agonist BAY60-6583 on basal synaptic transmission in the CA1 rat hippocampal slices. According to our previous results [15,38] BAY60-6583 (200 nM) did not significantly modify fEPSP slope during basal Schaffer collateral fiber stimulation (once every 15 s) in the CA1 rat hippocampus (Figure 3A,B, *n* = 11). fEPSP slope values were from 0.39 ± 0.03 mV/ms before to 0.40 ± 0.04 mV/ms after 20 min of applying the selective A_2B_AR agonist (see Table 1). Conversely, we demonstrated that the selective A_2A_AR agonist CGS21680 (50 nM) induced a modest, but significant, increase in fEPSPs slope (Figure 3C,D, *n* = 6) measured at the end of 20 min application. fEPSP slope values were from 0.49 ± 0.03 mV/ms before to 0.53 ± 0.03 mV/ms after 20 min of applying the selective A_2A_AR agonist (see Table 1). This result confirmed the involvement of A_2A_ARs in the CA1 basal synaptic transmission in accord to Lopes et al. (2002) [16]. The enhancement in basal synaptic transmission was prevented by the new dual A_2A_AR/A_2B_AR antagonist, P626 (200 nM, *n* = 7, Figure 3C,D). In particular, the fEPSP slope values were 0.48 ± 0.02 mV/ms in P626 alone and 0.48 ± 0.03 mV/ms in combination with CGS21680. When applied alone P626 did not modify, per se, basal synaptic transmission (see Table 1) nor PPF ratio (Figure S1).

**Figure 4:**
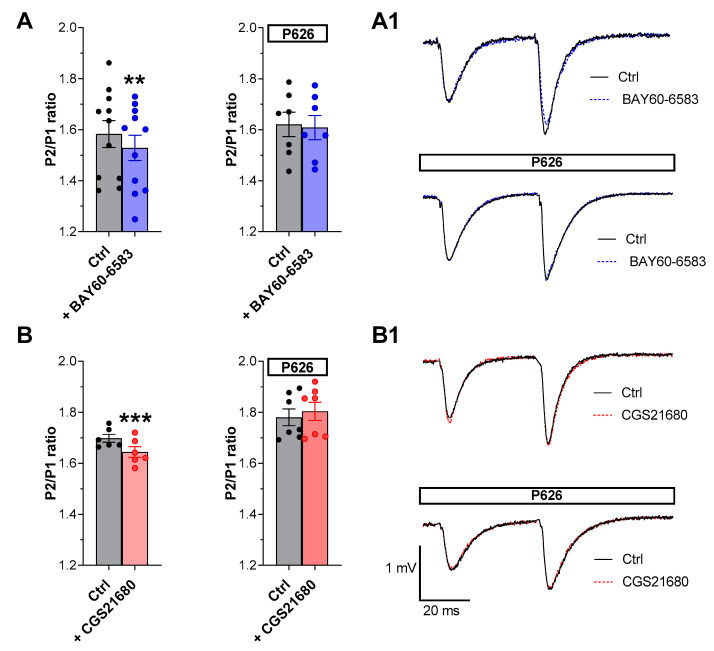
Effect of the new dual “A_2_ARs” antagonist, P626, on the inhibition of paired-pulse facilitation induced by the selective A_2B_AR or A_2A_AR agonists, BAY60-6583 or CGS21680, respectively, in the CA1 region of rat hippocampal slices. (**A**,**B**) Each graph shows pooled data (mean ± SEM) of paired-pulse facilitation (PPF), quantified as the ratio (P2/P1) between the slope of the fEPSP elicited by the second (P2) and the first (P1) stimuli. P2/P1 ratio is evaluated before (Ctrl), or 20 min after the application of BAY60-6583 ((**A**), *n* = 11 slices taken from 10 animals) or CGS21680 ((**B**), *n* = 6 slices taken from 5 animals) in the absence (left panel) or in the presence of the new dual antagonist P626 (*n* = 7 slices taken from 5 animals, right panels). (**A1**,**B1**) Representative traces of fEPSPs responses evoked by a PPF protocol recorded in different experimental conditions: (**A1**) before (black traces) and after (blue traces) the application of BAY60-6583 (200 nM) in the absence (upper panel) or in the presence (lower panel) of P626 (200 nM). (**B1**) before (black traces) and after (red traces) the application of CGS21680 (50 nM) in the absence (upper panel) or in the presence (lower panel) of P626 (200 nM). Note that the decrease of P2/P1 ratio induced by BAY60-6583 or CGS21680 was prevented in the presence of P626. ** *p* < 0.01, *** *p* < 0.001 vs. respective Ctrl, paired Student’s *t*-test.

In addition, we evaluated the effects of P626 in the absence or presence of BAY60-6583 or CGS21680 during the application of PPF, a paradigm of short-term synaptic plasticity. Following previous results [15,16] we confirmed that either BAY60-6583 or CGS21680 (Figure 4), significantly decreased the PPF ratio in CA1 rat hippocampal slices. Indeed, the P2/P1 ratio of fEPSP slope values, measured at the end of a 20 min application versus respective pre-drug baseline values, was reduced from 1.58 ± 0.05 in the absence to 1.52 ± 0.04 in the presence of 200 nM BAY60-6583 (Figure 4A, *n* = 11). Regarding the A_2A_AR agonist, P2/P1 ratio was from 1.70 ± 0.01 in the absence to 1.64 ± 0.02 in the presence of 50 nM CGS21680 (Figure 4B, *n* = 6). The inhibitory effects induced by both “A_2_ARs” agonists on PPF were completely prevented in the presence of 200 nM P626 (Figure 4A,B). Globally these results suggest that P626, antagonizing the reduction in PPF induced by the selective stimulation of “A_2_ARs”, may counteract the increase in neurotransmitter release elicited by either of the two receptor agonists.

### 3.2. P626 Delayed AD Onset Induced by Irreversible OGD in the CA1 Rat Hippocampus

**Figure 5:**
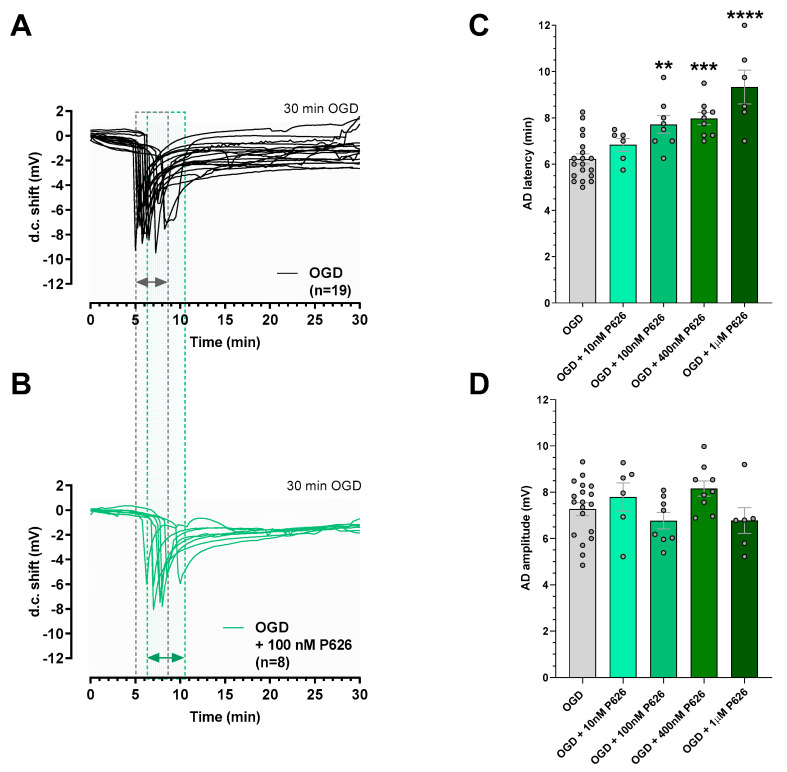
P626 delayed the appearance of anoxic depolarization (AD) induced by 30 min OGD without affecting AD amplitude in rat hippocampal slices. (**A**,**B**) The graphs show voltage traces of direct current (d.c) shifts recorded during 30 min OGD in untreated OGD slices ((**A**), *n* = 19 slices taken from 13 animals) or in the presence of 100 nM P626 ((**B**), *n* = 8 slices taken from 7 animals). Dotted lines and respective filled areas represent when the d.c shifts were recorded in the two conditions (grey–filled lines for the untreated OGD slices; green–filled lines for the P626–treated OGD slices). (**C**) Each column represents the mean ± SEM of AD latency recorded in CA1 hippocampal slices during 30 min OGD in different experimental groups. AD was measured from the beginning of the OGD insult. Note that 100 nM, 400 nM, and 1 µM P626 significantly delayed AD development. ** *p* < 0.01, *** *p* < 0.001, **** *p* < 0.0001 vs. OGD, one–way ANOVA followed by Bonferroni multiple comparison test. (**D**) Each column represents the mean ± SEM of AD amplitude recorded in the CA1 region during 30 min OGD. P626 10 nM: *n* = 6 slices taken from 5 animals; 400 nM: *n* = 9 slices taken from 8 animals; 1 µM: *n* = 6 slices taken from 5 animals.

The early phases of a hypoxic-ischemic insult are characterized by a significant increase in extracellular glutamate and adenosine levels [45]. The enhancement in glutamate release under pathological conditions contributes to excitotoxic damage [46]. In these experiments, we tested the new dual compound, P626, on neurotransmission before and after applying an irreversible, 30 min-long, OGD. This experimental protocol always elicited the appearance of AD, an unequivocal sign of tissue damage, and the irreversible failure of neurotransmission [30,38]. The experiments were conducted in the absence or the presence of different concentrations of P626 applied before, during, and 5 min after an ischemic-like episode. As illustrated in Figure 5, the appearance of AD in untreated OGD slices was recorded (Figure 5A,C), with a mean latency of 6.22 ± 0.21 min and a mean peak amplitude of 7.27 ± 0.28 mV (*n* = 19). The application of P626 was ineffective in modifying AD latency at the concentration of 10 nM (from 6.22 ± 0.21 min before to 6.83 ± 0.27 after drug application, Figure 5C, *n* = 6), while a significant AD delay started from the concentration of 100 nM (from 6.22 ± 0.21 min before to 7.72 ± 0.37 after drug application, Figure 5B,C, *n* = 8). When the OGD was applied in the presence of 400 nM or 1 µM P626, the d.c shift was also significantly delayed. The AD latency values were postponed to 7.98 ± 0.26 min in the presence of 400 nM P626 (Figure 5C, *n* = 9) and to 9.33 ± 0.72 min in the presence of 1 µM P626 (Figure 5C *n* = 6). Based on P626 affinity for all adenosine receptors, concentrations of P626 higher than 1 µM were not used since the compound could exert its effects by blocking all the adenosine receptor subtypes [44]. Finally, no difference in AD amplitude among all experimental groups was found (Figure 5D).

## 4. Discussion

In the present work, we provided the first evidence of the functional effects of the newly synthesized dual A_2A_AR/A_2B_AR antagonist, P626. This compound prevented the effects of A_2A_AR and/or A_2B_AR stimulation on short-term synaptic plasticity and during an ischemic-like insult in the CA1 region of rat hippocampal slices.

Multi-target compounds are designed to activate more than one cellular target simultaneously. Their use has increased in recent years, as these molecules offer the possibility to allow better pharmacokinetic and symptomatology control in various pathological conditions, by reducing side effects due to the administration of two different compounds [47].

To characterize the action/s of this innovative antagonist, we firstly demonstrated that P626 prevented the effects elicited by the selective A_2A_AR or A_2B_AR agonists, CGS21680 and BAY60-6583, respectively, on hippocampal neurotransmission either under basal condition or during PPF stimulation at Schaffer collateral-CA1 synapses. In particular, consistent with the literature (respectively: [15,16]), we confirmed, that CGS21680 significantly increased basal synaptic transmission, while BAY60-6583 did not show any effect. Of note, endogenous extracellular adenosine levels in acute hippocampal slices are estimated to be between 50 and 200 nM [8,45]. Hence, as the affinity of A_2A_ARs for the endogenous agonist is reported to be 20–300 nM, a submaximal A_2A_AR activation is already achieved before the CGS21680 application. Conversely, no activation of A_2B_ARs is expected to occur under physiological-like conditions because the affinity of this adenosine receptor for the endogenous ligand is over 20–30 µM [10]. Notably, the effect of CGS21680 on basal neurotransmission at CA1 synapses was antagonized by P626, thus demonstrating that this compound prevents A_2A_AR activation in the CA1 hippocampus.

The application of CGS21680 or BAY60-6583, during the PPF protocol, reduced P2/P1 ratio, which reflects a presynaptic increase in glutamate release at the hippocampal level [48,49]. These effects can be explained by the “residual Ca^2+^ hypothesis” of neurotransmitter release for inter-stimulus intervals lower than 500 ms (for review see: Zucker and Regehr 2002 [50]; Regher, 2012 [18]). In addition, the newly synthesized compound, P626, prevented the effects of the selective A_2A_AR or A_2B_AR agonist on PPF, proving once again that Gs-coupled adenosine receptors are involved in synaptic plasticity phenomena in the CA1 region, following data from Lopes et al., (2002) and Fusco et al., (2019) [15,16]. It is worth noting that one mechanism common to both A_2A_AR and A_2B_AR in the hippocampus is the downregulation of A_1_AR-mediated inhibition of synaptic transmission since PPF reduction by either A_2A_AR or A_2B_AR agonists is prevented by the selective A_1_AR antagonist DPCPX [15,20].

A_2A_ARs are known to be expressed on astrocytes [51,52], as well as on pre- and post-synaptic glutamatergic terminals of hippocampal neurons [12], where they can regulate synaptic plasticity [53,54] and neurotransmitter release [16]. Concerning the A_2B_AR, their expression in the central nervous system on glia and neurons is scarce but widespread if compared to A_2A_ARs [37,55] (for a review see: Coppi et al., 2020 [56]), and up to now evidence about their localization on hippocampal neurons is limited to presynaptic glutamatergic sites, where their activation is involved in the control of glutamate release [19]. The different localization of “A_2_ARs”, as well as the higher expression level of A_2A_ARs vs. A_2B_ARs [12,57], might explain the sole involvement of the A_2A_ARs in basal synaptic transmission, aside from the well-known inhibitory role of the A_1_AR in neurotransmission [58]. The facilitatory role of “A_2_ARs” is worthy of note as modification in neurotransmitter release probability, by affecting the filtering role of the hippocampus, influences the information-processing capabilities of the brain circuitry [49,59].

It is known that an increase in glutamate release plays different roles under physiological conditions; it facilitates neuronal excitability, synaptic plasticity, and coordination of neural networks. However, under pathological conditions (such as cerebral ischemia), this increase contributes to excitotoxic damage [46]. The early phases of a hypoxic-ischemic insult are characterized by a significant increase in extracellular glutamate levels, which triggers a hyper-activation of glutamate receptors, particularly NMDA subtype, production of reactive oxygen species, pathological increase in intracellular Ca^2+^, rapid decrease in ATP reserves and activation of various proteolytic enzymes [60,61,62]. Contemporarily to the glutamate increase, also the extracellular adenosine concentration significantly rises, as demonstrated both in vivo and in vitro experiments (for a review see: [45,63,64]). In these conditions, it is important to underline that A_2B_AR has a lower affinity for its endogenous ligand compared to the other adenosine receptor subtypes [65] which highlighted its selective involvement only in pathological conditions when extracellular adenosine concentrations reach micromolar levels. Therefore, A_2B_AR may represent a specific sensor of damage.

An OGD episode, which is an experimental condition that mimics the most common consequences of cerebral ischemia (embolic vessel occlusion), allows us to obtain highly valuable information in terms of the time course of the electrophysiological events, changes in membrane potential (i.e., AD) and synaptic transmission impairment [31,38,66]. As stated above, a pharmacological treatment that postpones the onset of AD could protect the penumbra, a brain region potentially salvageable after an ischemic-like insult [23,28,29,31,32]. The selective antagonism of A_2A_AR or A_2B_AR prevents or delays the AD onset induced by severe OGD in the CA1 region of the rat hippocampus. This mechanism reduces neuronal damage and astrocytic over-activation and stimulates survival pathways [30,38]. In this work, we tested the effects of the dual antagonist, P626, during 30 min OGD and we demonstrated that it could delay the AD onset induced by a severe OGD in the CA1 rat hippocampus. During the first minutes (2–3 min) of an OGD insult, adenosine concentration gradually increases activating principally the higher affinity A_1_AR and A_2A_AR subtypes. Then (after~4 min, see [6,8]), when the adenosine concentration reaches micromolar levels (between 10 and 30 micromolar), it is also able to activate the A_2B_AR subtype. Following these events, Fusco et al. (2019) demonstrated that the selective A_2B_AR antagonist, PSB603, did not modify OGD-induced fEPSP depression during the first 2 min of the ischemic-like insult, indicating that A_2B_ARs are not involved in the first phases of an ischemic episode [15]. This is also consistent with the extracellular adenosine levels measured over such a period, less than 5 μM [6,8,63,67], which are insufficient to activate the A_2B_ARs [10]. Therefore, during the first minutes after OGD, we presume that the new dual antagonist, P626, could exert its effect in delaying AD only by antagonizing the A_2A_AR subtype. Then, in the min following the OGD, when adenosine reaches a higher concentration, the overall effect of P626 could also be due to the block of A_2B_ARs. This condition is strengthened by the fact that, based on IC_50_ reported by Varano and colleagues [44], P626 apparently favors the block of A_2A_AR over A_2B_AR by about a factor of six. Thus, P626 could represent a favorable strategy for neuroprotection by a concurrent block of “A_2_ARs” subtypes during an acute ischemic insult. Moreover, given a translational clinical approach, we can speculate that the advantage of using the dual antagonist could be to bypass eventual differences in pharmacokinetic and side effects due to the administration of two different compounds. Finally, the simultaneous “A_2_ARs” blockade could have the presumed advantage of widening the therapeutic time window for efficacious post-stroke treatment.

## 5. Conclusions

In conclusion, the use of the novel dual A_2A_AR/A_2B_AR antagonist, P626, could represent a favorable strategy to achieve neuroprotection by a simultaneous block of “A_2_ARs” subtypes during an acute ischemic insult to prevent glutamate overload and expand the therapeutic time window in stroke patients.

## Figures and Tables

**Table 1:** Effects of the A_2A_AR and A_2B_AR ligands on fEPSP slope under basal conditions. Each value represents the mean ± SEM of fEPSP slope (expressed as mV/ms) obtained by the mean of twenty consecutive traces recorded immediately before (Ctrl) or after drug application (drugs). * *p* < 0.05 vs. respective Ctrl, paired Student’s *t*-test.

Treatment	*n*	Before (Ctrl) (mV/ms)	After (Drugs) (mV/ms)
200 nM BAY60-6583	11	0.39 ± 0.03	0.40 ± 0.04
50 nM CGS21680	6	0.49 ± 0.03	0.53 ± 0.03 *
200 nM P626	13	0.40 ± 0.03	0.40 ± 0.02

## Data Availability

The data that support the findings of this study are available from the corresponding author upon reasonable request.

## Supplementary Materials

The following supporting information can be downloaded at: https://www.mdpi.com/article/10.3390/biom13060894/s1, Figure S1: The new dual “A_2_ARs” antagonist, P626, did not affect basal synaptic transmission nor PPF ratio in the CA1 region of rat hippocampus.
