# Supplementary material for: Pharmacological Characterization of P626, a Novel Dual Adenosine A2A/A2B Receptor Antagonist, on Synaptic Plasticity and during an Ischemic-like Insult in CA1 Rat Hippocampus
Source: Biomolecules. 2023 May 27;13(6):894. doi: 10.3390/biom13060894 (PMC10296693; doi:10.3390/biom13060894)
Supplement: Supplementary file 1 [file biomolecules-13-00894-s001.zip › Caption Figure S1- Venturini et al Biomolecules.pdf]

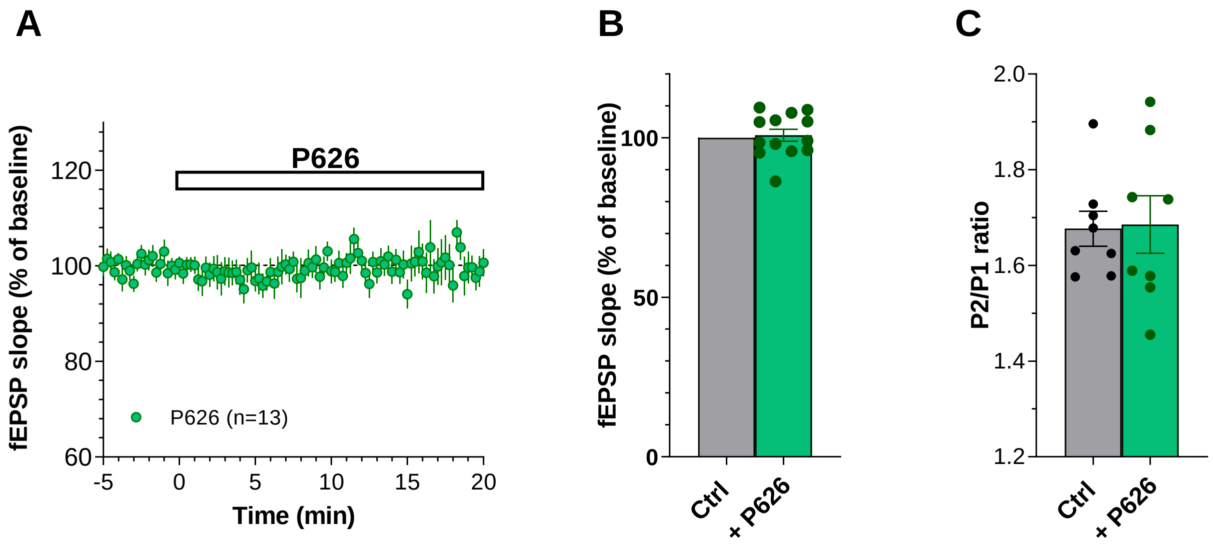

Figure S1. The new dual "A2ARs" antagonist, P626, did not affect basal synaptic transmission nor PPF ratio in the CA1 region of rat hippocampus. (A) Averaged time course of fEPSP slope under basal synaptic transmission, before and during the application of P626 (200 nM, n=13 slices taken from 8 animals). (B) Pooled data of fEPSP slope (mean ± SEM), expressed as percentage of respective baseline measured 5 min before and during the last 5 min of the application of P626. (C) The graph shows pooled data (mean ± SEM) of PPF, quantified as the ratio (P2/P1) between the slope of the fEPSP elicited by the second (P2) and the first (P1) stimuli. P2/P1 ratio is evaluated before (Ctrl) or 20 min after the application of P626 (200 nM, n=8). Paired columns refer to data collected from the same slice, before (Ctrl) or after the dual "A2ARs" antagonist, P626, application.
